# High Temperature Shear Horizontal Electromagnetic Acoustic Transducer for Guided Wave Inspection

**DOI:** 10.3390/s16040582

**Published:** 2016-04-22

**Authors:** Maria Kogia, Tat-Hean Gan, Wamadeva Balachandran, Makis Livadas, Vassilios Kappatos, Istvan Szabo, Abbas Mohimi, Andrew Round

**Affiliations:** 1 Brunel Innovation Centre (BIC), Brunel University, Cambridge CB21 6AL, UK; wamadeva.balachandran@brunel.ac.uk (W.B.); makis.livadas@brunel.ac.uk (M.L.); vassiliskappatos@gmail.com (V.K.); istvan.szabo@brunel.ac.uk (I.S.); abbas.mohimi@brunel.ac.uk (A.M.); andrew.round94@gmail.com (A.R.); 2 TWI Ltd., Granta Park, Great Abington, Cambridge CB21 6AL, UK

**Keywords:** Electromagnetic Acoustic Transducers, EMAT, Guided Wave Testing, high temperature inspection

## Abstract

Guided Wave Testing (GWT) using novel Electromagnetic Acoustic Transducers (EMATs) is proposed for the inspection of large structures operating at high temperatures. To date, high temperature EMATs have been developed only for thickness measurements and they are not suitable for GWT. A pair of water-cooled EMATs capable of exciting and receiving Shear Horizontal (SH_0_) waves for GWT with optimal high temperature properties (up to 500 °C) has been developed. Thermal and Computational Fluid Dynamic (CFD) simulations of the EMAT design have been performed and experimentally validated. The optimal thermal EMAT design, material selection and operating conditions were calculated. The EMAT was successfully tested regarding its thermal and GWT performance from ambient temperature to 500 °C.

## 1. Introduction

In nuclear, solar thermal and oil industries, many critical components, such as pipelines, pressure vessels, tanks and absorber tubes, operate at high temperatures and can suffer from creep, thermomechanical fatigue or hot corrosion [1,2,3]. As a result, they are likely to get damaged and their replacement and maintenance costs are high. Non-Destructive Testing (NDT) is required for their structural integrity assessment. The high temperatures, the access, size and structural complexity of the specimen can limit the number of NDT techniques that can be efficiently employed. Guided Wave Testing (GWT) can be applied to the inspection or monitoring of large structures from a single point. Guided waves can propagate large distances without significant attenuation; however, their velocity depends on both the geometry of the specimen and the excitation frequency—a phenomenon called dispersion, which complicates the interpretation of signals [4,5,6]. Shear Horizontal (SH_0_) or T(0,1) is not dispersive and cannot propagate in liquids, simplifying signal interpretation and inspection of structures carrying liquids like absorber tubes. GWT can also be used for the inspection of high temperature structures, but its efficiency is limited by the high-temperature performance of the transducers.

The dominant GWT transducer devices are piezoelectric. They are easy to use, have low cost and small size and do not require high driving voltages but do require physical contact with the specimen [7]. As a result, moving structures or components under vacuum cannot be inspected by piezoelectric transducers. Since the first high-temperature piezoelectric transducer appeared in 1940, several piezoelectric materials have been tested at high temperatures [8,9,10,11,12]. Lead Zirconate Titanate (PZT) is the most widely used material, operating efficiently up to 200 °C. Piezoelectric transducers can thermally decompose and lose oxygen at high temperatures [8,9,10,11,12]. Additionally, unlike GWT inspection, PZTs’ properties degrade significantly over time at high temperatures, making it unsuitable for GWT continuous monitoring [11,13]. When the Maximum Operating Temperature (MOT) of PZT is exceeded, the transducer permanently loses its piezoelectric property and requires a costly replacement [11,13].

Electromagnetic Acoustic Transducers (EMATs) are non-contact transducers, have simple designs, and can be used with GWT to inspect moving structures, high temperature objects or structures under vacuum, such as absorber tubes. They can excite/receive all types of wave (bulk, surface and guided) and require neither a viscous couplant nor a large force exerted to excite SH_0_ waves in the specimen [14,15,16,17,18,19,20]. They are not subject to skin effect and can be employed for the inspection of any electrically conductive material. They are more efficient on ferromagnetic specimens. However, they are power demanding and their ultrasonic performance varies with the material properties of the specimen. Their ultrasonic response is also lower than PZTs’—inspected lengths with EMATs are an order of magnitude smaller.

To date, high-temperature EMATs have been developed only for thickness measurements on metal blocks. Idris *et al.* have designed and tested a water-cooled EMAT for ultrasonic thickness measurements up to 1000 °C, comprising a Nd-F-B permanent magnet, Printed Circuit Board (PCB) spiral coil, a layer of mica and a cooling system [21]. Ultrasound was introduced to the specimen via a Q-switched Nd:YAG laser with the EMAT serving as detector. The EMAT was exposed to the heat source for the time needed to record a signal. Oil- and air-cooled EMATs have also been designed and reported to operate efficiently for thickness measurements at high temperatures for short periods [22,23,24]. Hernandez *et al.* have designed a pulsed EMAT for thickness measurement up to 600 °C for short time periods without any cooling [25]. The EMAT comprised a pulsed electromagnet designed for high temperatures and an alumina-encapsulated, copper, spiral coil. The EMAT was exposed to heat for times less than two minutes, depending on the temperature of the specimen.

The already-reported, high-temperature EMAT technology is not suitable for GWT. An EMAT that can be used in GWT and withstand high temperatures for either inspection or monitoring is still required to be designed and tested. This EMAT can be employed in oil and gas industry for high temperature GWT inspection of long pipelines or tanks, which carry flammable liquids, and their structural integrity assessment is of great importance. The high temperature GWT of moving or under vacuum structures can also be accomplished with the use of this EMAT.

This paper presents the theoretical and experimental evaluation of a Periodic Permanent Magnet (PPM) EMAT designed for GWT at high temperatures. The fundamental operating principles of EMATs, the effect of temperature on the EMAT performance and the new EMAT design are presented in the following section. Section 3 presents results obtained from thermal and Computational Fluid Dynamics (CFD) finite element analysis. In Section 4, the PPM EMAT prototype and preliminary high temperature tests are described. Section 5 shows the room and high-temperature ultrasonic response of the EMAT on steel and stainless steel plates. Conclusions from the experimental validation of the thermal models are also presented. Finally, the potential and limitations of the new high temperature EMAT design are summarized and future work is outlined.

## 2. EMAT Theory

EMATs comprise a permanent magnet (or electromagnet) for generating a static magnetic field, and a coil. An alternating current in the coil generates a dynamic magnetic field that induces eddy currents in the surface of the specimen. The eddy current electrons experience a force due to both their motion in the static field and the varying total field. If the specimen is a conductive, non-ferromagnet, the dominant force is the Lorentz force [14], equal to the product of the eddy current density and the total magnetic field.

(1) $$F_{L}=J_{e}\times \left(B_{\text{st}}+B_{\text{dyn}}\right)$$

where F_L_ is Lorentz force, J_e_ is the eddy current density, B_st_ is the static magnetic field and B_dyn_ refers to the dynamic magnetic field. In ferromagnetic specimens, such as steel and most nickel alloys, magnetostriction occurs and dominates [14]. Equations (2) and (3) give the coupled magnetostrictive relations; Equation (2) represents the direct magnetostrictive effect (Joule magnetostriction), where a strain is generated by a magnetic field, while Equation (3) gives the inverse magnetostriction (Vilarri effect), where stresses induce the field.

(2) $$\left\{\varepsilon \right\}=\left[S^{H}\right]\left\{\sigma \right\}+\left[d\right]\left\{H\right\}$$

(3) $$\left\{B\right\}=\left[\mu^{\sigma }\right]\left\{H\right\}+\left[d\right]\left\{\sigma \right\}$$

where ε is strain, S is elastic compliance at constant magnetic field, σ is the stress, d refers to the piezomagnetic strain constant (magneto-mechanical coupling coefficient), H is magnetic field strength, B is magnetic flux density and μ refers to the permeability of constant stress.

EMATs can generate all types of waves including longitudinal, shear, Lamb and guided waves. The coil configuration and the arrangement of magnets determine the wave mode and the frequency response of EMATs. Periodic Permanent Magnets (PPM) and magnetostrictive EMATs can excite/receive SH_0_. The former is made of an array of magnets of alternating magnetization directions, all normal to the sample surface. Straight wires are under the magnets and carry current of perpendicular direction to the magnetic field. This EMAT mainly generates Lorentz force regardless of the material properties of the specimen [14,15,16,17,18,19,20,26,27,28,29,30,31,32]. In magnetostrictive EMATs the static magnetic field and the excitation current have the same direction. The dynamic magnetic field is perpendicular to the static and only the direction of the total magnetic field changes. It is employed mainly on ferromagnetic materials and excites magnetostriction forces. Its performance is highly affected by the magnetic properties of the specimen [33,34,35,36]. This study focuses on the EMAT performance on both paramagnetic (stainless steel) and ferromagnetic (mild steel) materials. A PPM EMAT designed for high temperature GWT is analyzed in this paper.

If the effect of permanent magnets on the electrical connection between the EMAT and the specimen is ignored, the electric circuit of this connection can be modeled as an air core transformer [3,32]. The EMAT transmitter can be modeled as primary winding and the specimen as secondary. When the EMAT operates as a receiver, the primary winding represents the specimen and the EMAT receiver is the secondary winding. In either case, both windings experience electromagnetic losses due to their resistance, magnetic flux leakage and parasitic capacitance. These parameters are strongly related to the magnetic and electrical properties (permittivity, permeability, electrical conductivity and dielectric constant) of EMAT and specimen and can change with temperature. This causes variations in the efficiency of EMATs at high temperatures. As a result, the material selection for the designing of a high temperature EMAT is of great importance.

The electrical resistance of conductors changes with temperature, as Equation (4) shows.

(4) $$R\left(T\right)=R_{0}\left[1+a\left(Τ-{Τ}_{0}\right)\right]$$

where R_0_ is the resistance of a single turn coil, α refers to the temperature coefficient of resistance and T_0_ is room temperature. The temperature coefficient of copper is larger compared to that other low resistance conductors, such as silver, platinum, nickel and constantan, and copper also oxides at high temperatures. Silver, platinum, nickel and constantan also have high Curie temperature and can be used for the manufacturing of a high temperature coil. The MOT of constantan is 600 °C and its temperature coefficient is 0.00005 °C, however, its resistivity is 480 nΩm, while the resistivity of copper is 0.17 nΩm. The difference in resistivity between these two materials will affect the ultrasonic response of the EMAT. The amplitude of the signal received at room temperature will be smaller when constantan wire is used compared to copper. However, the constantan EMAT will be able to operate at higher temperatures compared to an EMAT with a copper coil [3]. Ceramics can also be used so that the coil will be thermally insulated. The ceramic encapsulation alters the coupling between the EMAT and the specimen, since the lift-off increases. The electrical properties of ceramics change with temperature and can result in variations in the electromagnetic losses and parasitic capacitance of EMAT at high temperatures [37].

Temperature rise also influences the performance of permanent magnets. The MOT of Nd-F-B is only 200 °C, restricting its use in high temperature EMAT technology. High Curie magnets can be used instead. SmCo and Alnico possess MOTs of 300 °C and 500 °C, respectively, however the magnetic fields they generate are weaker than Nd-F-B. Alnico also gets demagnetized easily in magnetic fields of opposite polarity and should not be employed in a PPM EMAT. EMATs designed for GWT should possess high magnetic capabilities, for maximum electromagnetic coupling with the specimen. The wave generated in the specimen must propagate long distances, requiring more energy to be introduced to the specimen compared to thickness measurements. Nd-F-B magnets are the strongest, but cannot be utilized at high temperatures without a cooling system so that their temperature remains below their MOT.

Specimen material properties, such as Young’s Modulus, Poisson ratio, and density, also change with temperature, affecting the velocity of the propagating wave. Young’s modulus and density decrease with increase in temperature, while Poisson ratio increases, resulting in a decrease in the velocity of SH_0_. Hence, as the temperature rises, any reflections received from the EMAT will shift in time and the temperature should be kept stable during the inspection or temperature compensation should be implemented for signal interpretation.

A racetrack coil, specifically designed to withstand high temperatures, was manufactured. Both Nd-F-B and SmCo magnets have been used. The EMAT was designed to accommodate a cooling system and the thermal response of several designs was investigated through transient thermal simulations. The EMAT design with the best thermal properties was manufactured. The optimal operating conditions for the cooling system were confirmed using CFD simulations.

## 3. Thermal Finite Element Analysis

The EMAT was designed so that the temperature of the coil and the magnets remain below their MOT. Given that the coil is directly exposed to heat and its spacing from the magnets is usually less than 1 mm, its cooling becomes a challenge. No cooling system can be easily designed to efficiently cool down the coil without affecting the specimen. Conductive materials, with MOT higher than 500 °C, can be used for the manufacturing of the coil like constantan. Thermal insulating materials should be also added to the coil to impede the heat transfer from the specimen. However, EMATs are very sensitive to lift-off variations, since the electromagnetic coupling strength between the EMAT and the specimen decreases with lift-off. Consequently, the thickness of any material added to the coil should be as small as possible so that both the coil will be efficiently protected from the heat and the electromagnetic coupling will be maximized. The optimum thickness of the thermal insulation was investigated via thermal simulations.

The temperature of the magnets should be maintained below 200 °C, so that both types of magnets can be used. Thermal insulation of the coil will increase the time required for energy to be transmitted to the magnets but excess thermal energy can lead them to break down. The heat must therefore be removed via a cooling system designed into the EMAT. Optimal flow velocity and inlet temperature of the coolant were identified via CFD simulations. The effect of the cooling medium on the performance of the cooling chamber was also investigated and a comparison of the thermal efficiency of water and oil performed.

### 3.1. Transient Thermal Simulations

**Figure 1:**
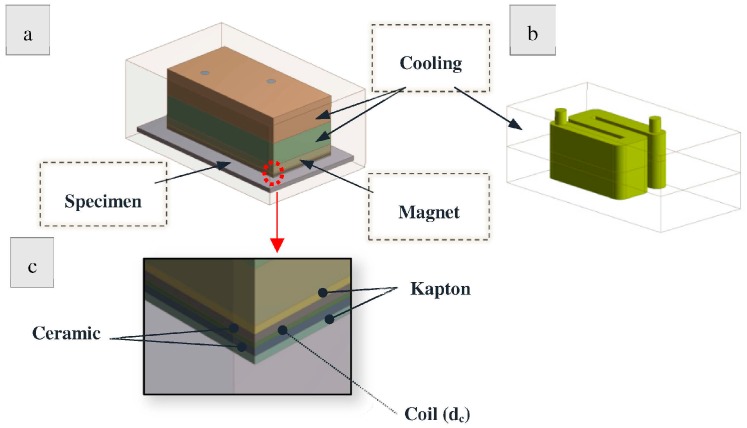
Thermal EMAT model (**a**) entire EMAT design; (**b**) Cooling chamber; (**c**) Coil.

Finite Element Analysis was used for the thermal evaluation of a simplified EMAT design. Only the main parts of the EMAT were modeled, including the cooling, the magnets/magnet holder and the coil structure. The housing of the EMAT was made of brass, which possesses high thermal conductivity and melting point compared to aluminum and stainless steel transducer housings. A rectangular box of 3 mm thickness of 316 L stainless steel was modeled as the specimen/heat source. Figure 1 shows the geometry analyzed in the Ansys Transient Thermal Module model. In all simulations, the EMAT was in 100% contact with the specimen so the results correspond to the severest thermal conditions. The EMAT components are denoted in Figure 1a and the cooling chamber in Figure 1b. A detailed image of the coil is given in Figure 1c. The coil is made of constantan and is encapsulated in alumina between two layers of Kapton.

**Figure 2:**
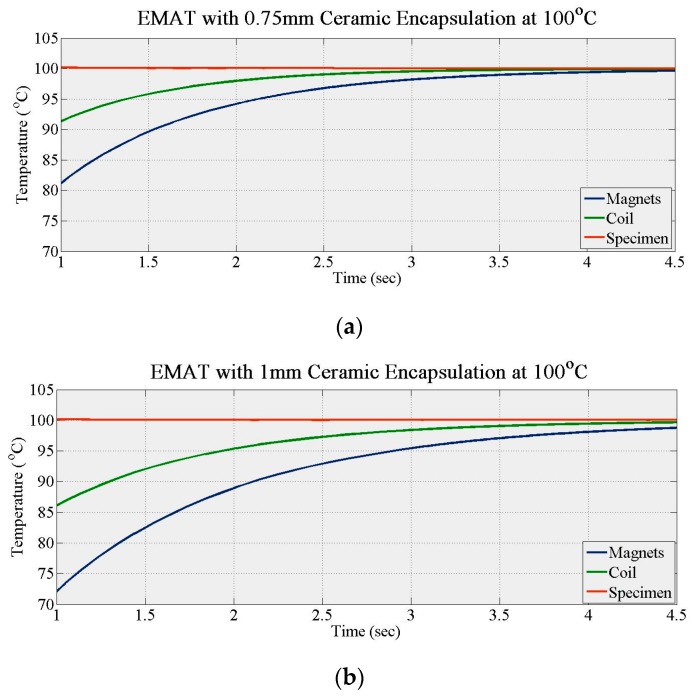
Temperature gradient of alumina encapsulated coil (**a**) 0.75 mm alumina thickness; (**b**) 1 mm alumina thickness.

**Figure 3:**
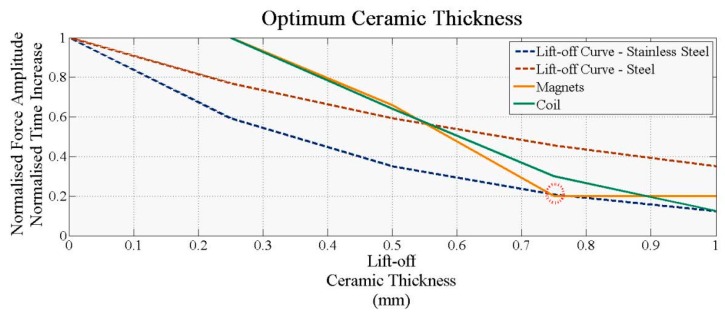
Optimum ceramic thickness graph.

During the first set of simulations, the temperature of the specimen was set to 100 °C with no active cooling. No Kapton encapsulated the coil whose diameter/thickness (d_c_) was fixed at 0.4 mm. The thickness of the alumina layers (t_c_) was increased gradually in simulations from zero with a step of 0.25 mm until the thermal response of the EMAT essentially plateaued. The maximum thickness was 1 mm due to lift-off limitations. The time needed for the coil (t_max_coil_) and magnets (t_max_magnets_) temperature to maximize did not increase significantly for alumina thickness exceeding 0.75 mm, as Figure 2 and Figure 3 show. Figure 2a,b show the temperature increase of the coil and magnets for 0.75 and 1 mm thick alumina layers. In Figure 3, the green and orange solid lines show how the normalized time required for the coil and the magnets temperature to reach their maximum (t_norm_coil_, t_norm_magnets_) converges with ceramic thickness. Equation (5) gives t_norm_coil_ and t_norm_magnets_ is calculated in the same manner, where *i* is the step in ceramic thickness increase. The blue and red dashed lines in Figure 3 present how the normalized Lorentz force amplitude drops with lift-off for stainless steel and steel, respectively, based on Equations (6) and (7), respectively, where G is the lift-off and D refers to the pitch of the PPM EMAT.

(5) $$t_{\text{norm\_coil}}=\frac{t_{\text{max\_coil\_i}}-t_{\text{max\_coil\_i+1}}}{t_{\text{max\_coil\_i}}},\;i=1,2,3$$

(6) $$F_{\text{L\_stainless steel}}=e^{\frac{-12\pi }{GD}}$$

(7) $$F_{\text{L\_steel}}=e^{\frac{-4\pi }{GD}}$$

Figure 3 shows that t_norm_magnets_ plateaus after 0.75 mm ceramic thickness. Consequently, any further ceramic thickness increase will not improve significantly the thermal response of EMAT over time. The ceramic thickness can also affect the ultrasonic response of EMAT, since it increases its lift-off. When the ceramic thickness is 0.75 mm, Lorentz force decreases to 20% and 45% of its maximum for stainless steel and steel, respectively. When the ceramic thickness increases to 1 mm, Lorentz force in stainless steel is less than 20% of its maximum, making the EMAT inefficient for GWT. Hence, the minimum thickness of each alumina layer should be chosen as 0.75 mm. Regardless of the ceramic thickness, the time needed for the EMAT components to reach maximum temperature is of the order of only seconds. Hence, additional thermal insulation is needed to extend operation time.

**Figure 4:**
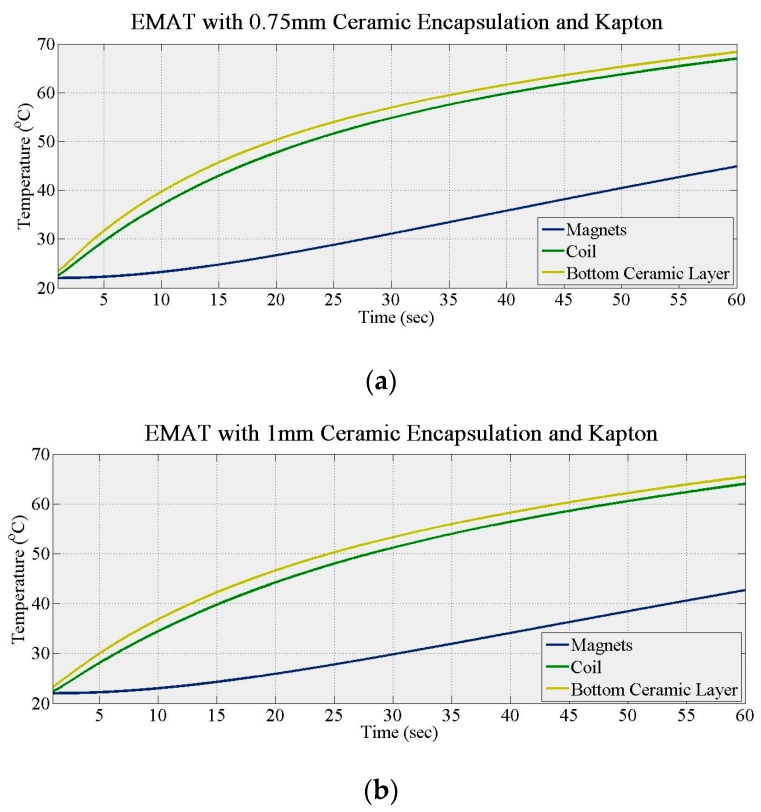
Temperature gradient of Kapton and alumina encapsulated coil (**a**) 0.75 mm alumina thickness; (**b**) 1 mm alumina thickness.

Further simulations were carried out with two Kapton layers in place. The alumina thickness increased from 0.75 mm to 1 mm in 0.25 mm steps with a Kapton layer (t_k_) of 1 mm and a specimen held at 100 °C. Figure 4a (0.75 mm alumina) and Figure 4b (1.0 mm alumina) show the temperature increase of the bottom ceramic layer, the coil and the magnets for the fully encapsulated EMAT. The response of all three components did not change significantly with the ceramic layer, indicating that a 0.75 mm thick alumina encapsulation is suitable for this design. However, the maximum temperature of the affected; both the first and second derivatives of the magnet responses change compared to Figure 2, explained by the double insulation of the magnet compared to the coil, with two layers of alumina and Kapton inhibiting heat transfer. When both Kapton and 0.75 mm thick alumina encapsulation are used and the EMAT heated for 1 min, the temperature of the coil and the magnets reaches only 67 °C and 45 °C, respectively. Thus, the higher temperature response of this design must be investigated.

In the final simulations, the EMAT design (coil encapsulated with 0.75 mm thick alumina and two layers of Kapton) was tested up to 500 °C. No active cooling was used and the temperature of the specimen increased from 100 °C to 500 °C. Table 1 summarizes the temperature of the coil and the magnets with the EMAT heated for 1 min. The results show that alumina and Kapton can be used for thermal insulation of the coil and magnets, since the temperature of the EMAT components remains well below their MOT. Nevertheless, these results indicate that the design will withstand high temperatures only for a short period of time and further CFD simulations must be carried out.

### 3.2. CFD Simulations of Water Cooled EMAT

**Figure 5:**
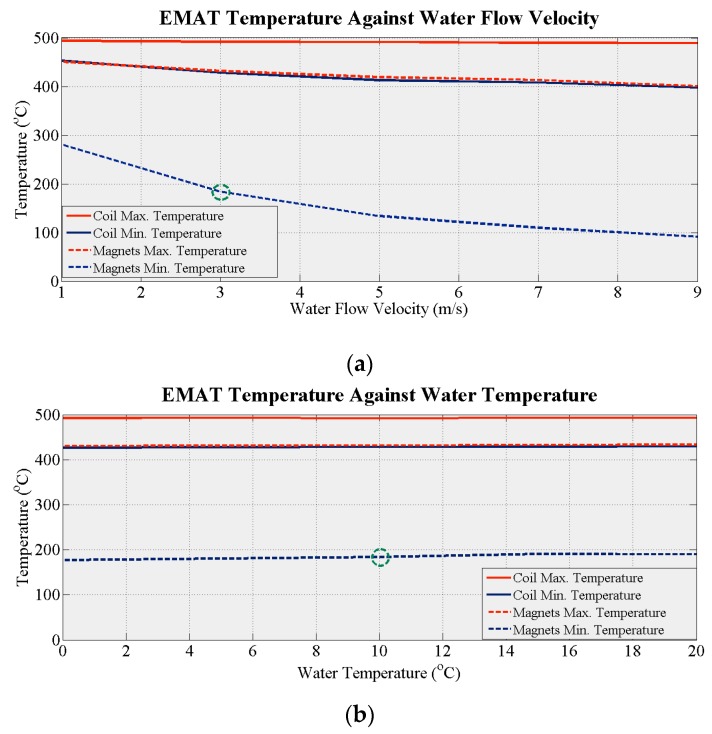
(**a**) EMAT temperature against coolant flow velocity; (**b**) EMAT temperature against coolant temperature.

The thermal behavior of the proposed design has been also simulated with active cooling to determine the optimum cooling system parameters. In the first set of simulations, the optimum coolant flow velocity was evaluated. The duration of the coolant in the cooling chamber exposed to heat affects the amount of thermal energy it absorbs. The temperature of the coolant increases with time, reducing its temperature difference with the heat sink until eventually this is too small to dissipate sufficient heat. The optimum flow velocity must therefore be calculated. In these simulations, water with inlet temperature of 10 °C was used as coolant. The density, specific heat capacity, thermal conductivity and viscosity of water are, respectively, 998 kg/m^3^, 4182 J/kg·K, 0.6 W/m·K and 0.001 Pa/s. The temperature of the specimen was held at 500 °C and the EMAT was in total contact with the specimen. The water flow velocity was increased in the simulations from 1 m/s to 9 m/s in steps of 2 m/s. Figure 5a demonstrates how the maximum and minimum temperature of the coil and the magnets drop with increasing flow velocity. The minimum temperature of the magnets decreases dramatically. It drops below 200 °C at a flow velocity of only 3 m/s showing that flow velocity has a major influence on EMAT efficiency. The optimum flow velocity for this system in terms of power requirement is 3 m/s since higher flow velocities would require more electrical power.

A parameter that could affect the efficiency of the system is the temperature of the coolant entering the cooling area. In further simulations, the water flow velocity was kept at 3 m/s and its temperature increased from 0 °C to 20 °C in steps of 5 °C. The balance of the components was held at 25 °C. Figure 5b shows how the maximum and the minimum temperature of the coil and the magnets change with the water temperature. No significant changes are observed in any of the four temperatures as the coolant temperature increases. Thus, this parameter cannot be used to control the performance of the cooling system. An optimum coolant temperature of 10 °C can be freely chosen since the minimum temperature of the magnets is below 200 °C and no additional equipment is required to cool the water.

**Figure 6:**
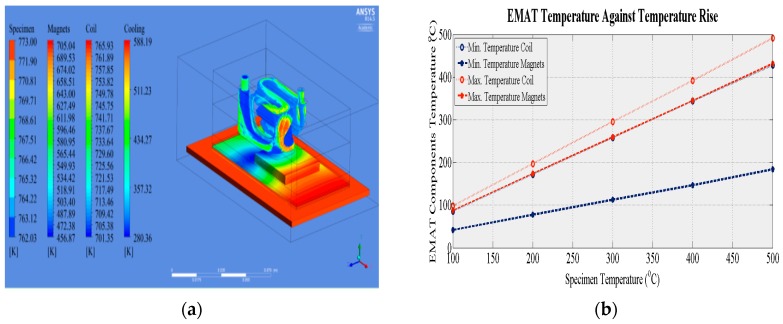
Water cooled EMAT (**a**) temperature gradient of EMAT components at 500 °C; (**b**) temperature of EMAT components against temperature rise. The temperature range of the magnets, coil and the cooling medium are depicted in figure a.

Figure 6a shows the temperature gradient of the main EMAT components when the EMAT is heated for 10 s. It is apparent that the size and position of the cooling chamber affects the temperature gradient of the coil and the magnets. The area below the cooling chamber is efficiently cooled while areas further away from the cooling chamber and closer to the cooling outlet experience higher temperatures. However, the size and position of the cooling chamber was directly affected by the Bayonet Neil Concelman (BNC) connector position. Space was needed for the electrical components of the EMAT to be safely positioned without the overall size of the EMAT exceeding 100 mm. A design with wider cooling chamber could cool the critical EMAT components more efficiently but may result in a bulkier EMAT. Figure 6b summarizes the results obtained when the aforementioned model was used for the evaluation at temperatures from 100 °C to 500 °C in steps of 100 °C. In all cases, the minimum temperature of the main two EMAT components does not exceed their MOT, indicating that this EMAT design under the specified conditions can withstand high temperatures. Nevertheless, the maximum temperature of the magnets exceeds their MOT when the specimen temperature exceeds 250 °C, and the maximum water temperature exceeds 100 °C. However, these temperatures occur in a small area and the short time required for the water to circulate in the cooling chamber means the water will not evaporate.

### 3.3. CFD Simulations of Oil Cooled EMAT

The material properties of the cooling medium—heat capacity, thermal conductivity, freezing point and viscosity—affect the performance of the cooling system, its thermal efficiency and limitations. Water and oil have been widely used as coolants for transformers and EMATs and a thermal analysis was performed for an oil-cooled EMAT. The density, specific heat capacity, thermal conductivity and viscosity of the oil used in the model are respectively 890 kg/m^3^, 1860 J/kg·K, 0.126 W/m·K and 0.06 Pa/s. Flow velocity was 3 m/s and the temperature of oil as it entered the cooling chamber was 10 °C while the rest of the EMAT components were at 25 °C. The temperature of the specimen was held at 500 °C with the EMAT in contact. Under these conditions the oil did not cool the magnets as efficiently as water; the minimum temperature of the magnets exceeded 200 °C. However, optimum operating parameters change with the coolant and since it was simulated under the optimum conditions for water, the thermal performance of the oil-cooled EMAT was low. The temperature of oil can be below zero, if a refrigerator is used, however this results in complicated and costly instrumentation. The oil cooled EMAT design was simulated again with an oil temperature of −10 °C and the flow velocity was set to 3 m/s and then 9 m/s. Table 2 summarizes the results retrieved from the three case studies for the oil cooled EMAT; neither flow velocity nor temperature significantly improve the oil system’s efficiency. 

Oil has a smaller heat capacity and thermal conductivity than water and less time is required for its temperature to increase. The viscosity of oil also impedes its circulation inside the cooling chamber. Hence, the temperature difference between coil and heat sink is smaller compared to that with water-cooling, resulting in lower efficiency. The cooling system must be re-designed to improve the thermal response of the oil cooled EMAT. Magnets and coil can be in a single chamber through which cooling oil flows. Oil is a minor fire hazard. It may also attenuate and alter the magnetic field and its dielectric constant alters with temperature. Further research with oil is necessary. Water is safer but its higher freezing point limits cooling efficiency. Consequently a water-cooled EMAT with Kapton and ceramic encapsulated coil can withstand high temperatures for short period based on theoretical analysis and can potentially be used for high temperature inspection. Experimental validation is still required.

## 4. Experimental Procedure

### 4.1. EMAT Development

**Figure 7:**
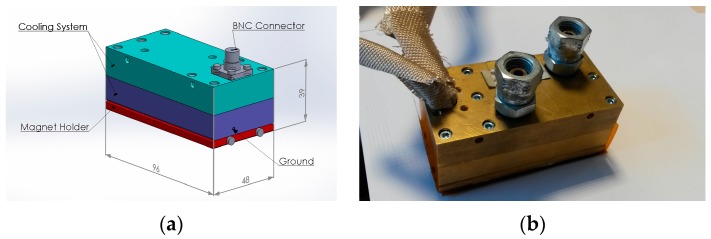
(**a**) EMAT design; (**b**) EMAT prototype.

**Figure 8:**
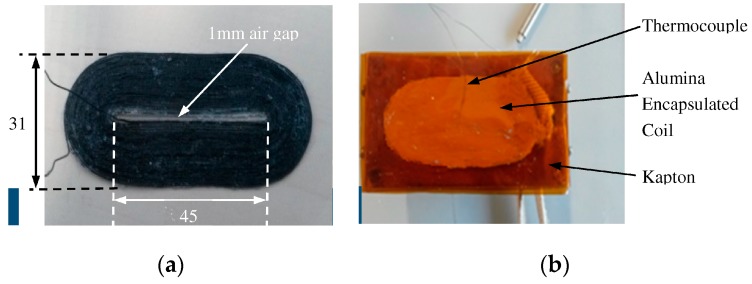
(**a**) Constantan Coil; (**b**) Alumina and Kapton encapsulated coil (in mm).

The new EMAT was manufactured and is shown in Figure 7. All housing components are made of brass and both Nd-F-B and SmCo magnets have been used and arranged in a PPM configuration. The CAD design is shown in Figure 7a,b shows the EMAT prototype. Figure 8a shows the racetrack coil without either alumina or Kapton encapsulation. Figure 8b depicts the bottom view of the constantan coil, as it is encapsulated in alumina within two Kapton layers and a thermocouple. The wire diameter is 0.4 mm and the thickness of the each alumina layer is 0.8 mm. The Kapton layer is 0.1 mm thick and its melting point is 400 °C. If a single Kapton layer of this thickness is not thermally efficient for impeding the heat transfer, more layers can be added to the structure until the total thickness of the Kapton encapsulation is 1 mm at each side. Care must be taken if the temperature exceeds 350 °C, since the melting point of Kapton is 400 °C. The EMAT was designed so that the coil can be replaced easily and coils of different shape and/or material can be connected to the rest of the transducer. The coil ends was also extended so that a commercial BNC connector, of which MOT is below 500 °C, can be used.

### 4.2. Experimental Setup

The EMATs were tested regarding their ultrasonic and thermal performance for inspection and an experimental validation of the thermal model was accomplished. They were tested on stainless steel and steel plate at room and high temperatures and both types of magnets were tested. The ultrasonic potentials of this EMAT design for GWT inspection on both paramagnetic and ferromagnetic materials were investigated at room and high temperatures (500 °C).

**Figure 9:**
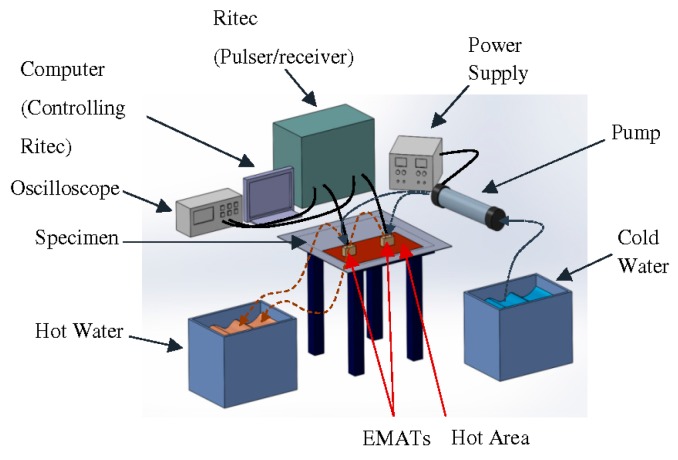
Experimental setup.

The experimental setup is shown in Figure 9. A pair of EMATs was attached to the specimen with no lift-off. They were employed in a pitch-catch configuration with 30 cm between them and 40 and 50 cm between the transmitter and the edge of the steel and the stainless steel plate respectively. A square, 316 Ti stainless steel plate of 1.25 m edge length and 3 mm thickness and a square, mild steel plate of 1 m edge length and 3 mm thickness were used as specimens.

The EMAT transmitter was driven by a Ritec RAM 5000 SNAP pulser / receiver unit (RITEC Inc., Warwick, RI, USA) with an 8 cycle pulse of 256 kHz. The excitation pulse was Hanning windowed when the EMATs were tested on stainless steel, while no Hanning window was applied on the excitation pulse when steel was tested. The EMAT receiver was also connected to a Ritec so the raw signal could be amplified with 80 dB gain and filtered with high and low-pass filters of 10 kHz and 20 MHz cut-off frequencies respectively. The filtered signal was collected, averaged and recorded with a 2-channel Agilent oscilloscope (Keysight Technologies Inc., Santa Rosa, CA, USA). A pump (D5 Photon 170 Pump Combo) was used for circulating the water in the two EMATs with a flow velocity of 3 m/s while the temperature of the cold water was 10 °C. The specimen was heated up by a heat treatment module manufactured by Stork (STORK, Utrecht, The Netherlands); the module was connected to four ceramic pads, which were composed of high grade sintered alumina ceramic beads and nickel chrome core wires. The pads were driven with high current by the module and the thermal energy generated via the nickel chrome wires was transmitted to the specimen. The temperature of the specimen, the EMAT coil, the magnets and the water outlet of the cooling chamber were monitored by a 4-channel thermo-logger. The thermocouples attached to the coil and the magnets were placed on the area underneath the cooling chamber where the temperature is lower based on the simulations.

The thermal conductivities of both stainless steel and steel are low (16 and 50 W/m·K respectively at room temperature) and thermal energy introduced to a certain area of the plates cannot readily diffuse throughout the entire specimen volume. Only the area underneath the heating pads can be heated efficiently to 500 °C. As soon as the heating pads were removed from the plates, the temperature of the area of inspection decreased quickly. Testing done at high temperatures for only short times and the experiments were conducted three times for each EMAT. The temperature of the heating pads was increased from ambient to 700 °C and the maximum temperature of the area below the pads was 600 °C. With the pads removed and the EMATs placed with zero lift-off on the defect-free, heated area, the specimen temperature was 500 °C. The ultrasonic response and the temperature of the coil and magnets were recorded from 500 °C down to ambient temperature in steps of 50 °C.

## 5. Experimental Results and Discussion

**Figure 10:**
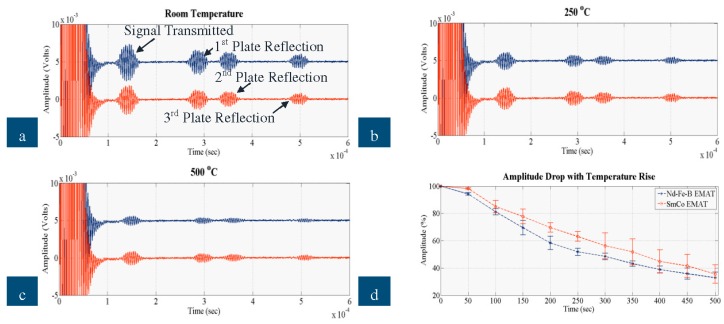
Signal received by Nd-F-B and SmCo EMAT on steel at (**a**) room temperature; (**b**) 250 °C; (**c**) 500 °C; (**d**) amplitude drop against temperature rise.

**Figure 11:**
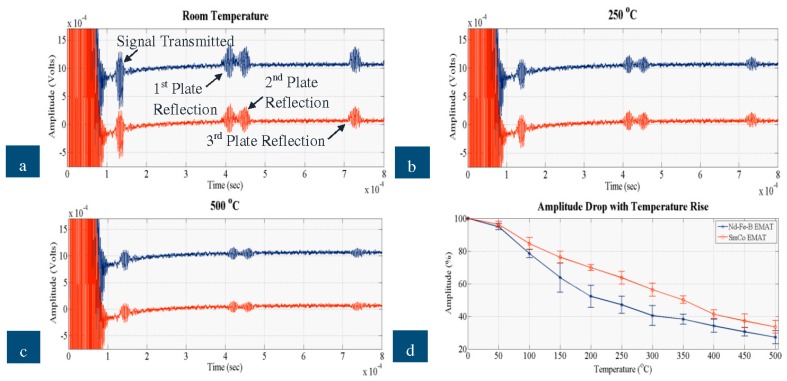
Signal received by Nd-F-B and SmCo EMAT on stainless steel at (**a**) room temperature; (**b**) 250 °C; (**c**) 500 °C; (**d**) amplitude drop against temperature rise.

The signals received when a defect free area was inspected by the new EMATs on the stainless steel and steel plates are shown in the following figures. In each graph, the blue signal corresponds to the Nd-F-B EMAT and the red signal to the SmCo EMAT and all of them show the signal transmitted and the three reflections coming from the edges of the plate. Figure 10a–c illustrate the ultrasonic signals recorded when the EMAT was tested on the steel plate at room temperature, 250 °C and 500 °C, respectively, and Figure 10d depicts how the amplitude of the signal received decreases with temperature for all the three experimental sets. Figure 11 shows the signals received when the EMATs were tested on the stainless steel plate. Figure 11a–c shows the ultrasonic signal at room temperature, 250 °C and 500 °C and Figure 11d presents how the amplitude of the signal received decreased with temperature on stainless steel. 

Based on the dispersion curves calculated for the stainless steel and steel plates, the velocities of SH_0_ equal 3080 and 3203 m/s, respectively. Thus, the Time of Flight (ToF) of the reflections shown in the graphs match the velocity of SH_0_; ToF of all four reflections is increased by 30 μs pulse duration. The proposed EMAT configuration, thus, has adequate wave purity characteristic for the excitation/reception of SH_0_ and for GWT of structures carrying liquids. As expected the EMAT performs better on steel than on stainless steel due to the stronger electromagnetic coupling. The amplitude of the signal received for steel is an order of magnitude larger than stainless steel. Nevertheless, this EMAT design is able to excite and receive guided waves on stainless steel at temperatures up to 500 °C. Compared to the high temperature ultrasonic response of a conventional, room temperature PPM EMAT made of Nd-F-B magnets and copper coil studied in detail in [3], the new EMAT can be used for GWT up to 500 °C, while the conventional can hardly be employed up to 100 °C. The cooling system, the material selection (constantan coil, SmCo magnets) and the optimum operating conditions can significantly improve the EMAT ultrasonic response at high temperatures. The new EMAT could be utilized for inspection of absorber tubes and other conducting, paramagnetic, high-temperature objects. All four reflections also shifted in time as temperature increased, as expected.

On both steel and stainless steel, the SmCo EMAT is more efficient with respect to amplitude and temperature, compared to Nd-F-B. At room temperature the amplitude of the Nd-F-B EMAT is larger than that received by the SmCo. Regardless of the material tested, the performance of the Nd-F-B EMAT fluctuates slightly from 200 °C to 350 °C, indicating that over this range the temperature of the magnets must have reached the MOT of Nd-F-B. The amplitude drop with Nd-F-B is slightly larger when stainless steel is inspected due to weaker electromagnetic coupling with the specimen. The amplitude of the signal received with SmCo EMAT drops at a constant rate as temperature rises, especially on stainless steel, and its amplitude drop over the entire temperature range is smaller than with Nd-F-B. All this indicates that the temperature of the magnets is close to the MOT of Nd-F-B, especially from 200 °C to 500 °C, resulting in stable performance of the SmCo EMAT and the fluctuating performance of the Nd-F-B EMAT. The cooling system appears efficient for both types of magnet, since ultrasonic signals were obtained over the entire temperature range and the SNR did not deteriorate with temperature rise. Nevertheless, SmCo EMAT can better withstand high temperatures and its amplitude is larger at high temperatures compared to Nd-F-B.

**Figure 12:**
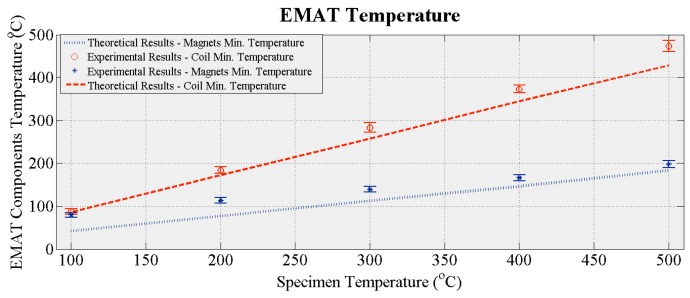
Measured temperature of EMAT coil and magnets *vs.* temperature.

Figure 12 shows how the temperature of the EMAT coil and magnets changes with the temperature of the specimen. The red dashed and blue dotted curves denote the calculated minimum temperature of the coil and the magnets from the CFD simulations presented in Section 3.2 and shown in Figure 6b. The red circles and blue stars are the measured temperatures of the coil and the magnets, the error bars depicting the variation in the three experimental sets. The experimental values agree with theory, excepting the measured temperature of the magnets at 100 °C, which is approximately twice the theoretical value. During the experiments the specimen temperature decreased gradually after reaching 500 °C, while in the simulations the temperature was held at a certain target value. Therefore, it is likely the EMAT and its housing maintain a portion of the thermal energy absorbed at 500 °C until dissipated by the cooling system. The time needed for the maximum energy to be dissipated is larger than the time needed for the temperature of the specimen to decrease to 100 °C. Therefore, the temperature of the EMAT did not drop with the calculated rate and the largest discrepancy between theoretical and experimental values is observed at 100 °C. Nevertheless, the thermal model can be used for the analysis of EMATs when the temperature of the specimen remains constant; during online inspection/monitoring the temperature of the specimen usually stays constant or changes only slightly.

## 6. Conclusions and Future Work

GWT using EMATs can be used for inspection of high temperature, complex structures without contact. A water-cooled EMAT with a Kapton and alumina encapsulated, constantan coil was theoretically and experimentally evaluated for GWT at room and high temperatures. Thermal and CFD simulations were carried out to optimize EMAT design, materials and operating conditions. Both Nd-F-B and SmCo magnets were used during the experiments and the EMATs were tested on both steel and stainless steel on a defect free area for short times. Both EMATs excited and received SH_0_ successfully in all cases validating its use in inspection of high-temperature structures.

Further experiments on the guided wave performance of this EMAT over long periods of time will be conducted and a detailed characterization at both room and high temperature made. Lift-off limitations and power supply requirements will be further investigated. The effects of temperature on the EMAT impedance and electrical circuit characteristics will be further evaluated. 

## Figures and Tables

**Table 1:** Temperature of components of final Electromagnetic Acoustic Transducers (EMAT) design.

Specimen	Temp./°C				
	100	200	300	400	500
Coil	66.4	123.4	180.3	236.7	294.4
Magnets	43.9	72.1	100	129.2	156.5

**Table 2:** Temperature of oil cooled EMAT against flow velocity and oil temperature.

Medium	Flow Velocity	Coolant Temperature	Coil Max. Temperature	Coil Min. Temperature	Magnets Max. Temperature	Magnets Min. Temperature
	m/s	°C	°C	°C	°C	°C
Oil	3	10	497	482.9	480	421.1
Oil	3	–10	496.8	482.2	479.1	417.9
Oil	9	–10	496.1	480.4	477.2	413.4
Water	3	10	491.8	428.1	431.8	183.6
